# Optimization of Ultrasonic-Assisted Extraction and Radical-Scavenging Capacity of Phenols and Flavonoids from *Clerodendrum cyrtophyllum* Turcz Leaves

**DOI:** 10.1371/journal.pone.0068392

**Published:** 2013-07-16

**Authors:** Jing Zhou, Xiaoxue Zheng, Qi Yang, Zhenyi Liang, Donghai Li, Xiaobo Yang, Jing Xu

**Affiliations:** 1 Key Laboratory of Protection and Development Utilization of Tropical Crop Germplasm Resources, Ministry of Education, College of Material and Chemical Engineering, Hainan University, Haikou, P. R. China; 2 Institute of Functional Biomolecules, State Key Laboratory of Pharmaceutical Biotechnology, Nanjing University, Nanjing, P. R. China; University of Sassari, Italy

## Abstract

Ultrasonic-assisted extraction (UAE) was developed to extract phenolic and flavonoid antioxidants from *Clerodendrum cyrtophyllum* Turcz leaves. The optimal experimental parameters for antioxidant extraction from *C. cyrtophyllum* leaves were measured using single-factor experimentation combined with response surface methodology (RSM). Total phenolic content (TPC) and total flavonoid content (TFC) assays were used to quantify antioxidant compounds. Next, antioxidant radical scavenging capacity was measured using 2,2′-diphenyl-1-picrylhydrazyl (DPPH) and 2,2′ -azino-bis(3-ethylbenzothiazoline-6-sulphonicacid) (ABTS) radicals. Optimized extraction conditions for UAE from *C. cyrtophyllum* leaves were as follows: 60.9% ethanol, 85.4 min, and 63.3°C for maximal TPC extraction (16.8±0.2 mg GAE/g DW); 67.7% ethanol, 82.9 min, and 63.0°C for maximal TFC extraction (49.3±0.4 mg RT/g DW); 48.8% ethanol, 85.1 min, and 63.9°C for maximal DPPH radical-scavenging capacity (86.8±0.2%); and 50.6% ethanol, 81.3 min, and 63.4°C for maximal ABTS radical-scavenging capacity (92.9±0.5%). Ethanol concentration was the most important factor in the extraction process. Our work offers optimal extraction conditions for *C. cyrtophyllum* as a potential source of natural antioxidants.

## Introduction

Studies in the literature have long reported that free radicals are important in the progression of various diseases implicated with aging, such as cancer, cardiovascular disease, emphysema, cirrhosis, arthritis, diabetes mellitus, cataracts, inflammation, and brain disorders [1]. Fortunately, free radical formation can be reduced by antioxidants, which scavenge and neutralize free radicals [2]. Synthetic antioxidants are frequently used in food and pharmaceutical products, but their use raises consumer questions regarding side effects and potential toxicities [3]. Thus, safe, natural antioxidant alternatives are desired to protect the human body from oxidative stress and retard potential chronic diseases of aging. Specifically, antioxidants with plant origins are of considerable interest [4]. Certain phytochemicals, especially plant phenolics and flavonoids, may be potential antioxidants with chemopreventive effects. The antioxidant activity of phenolics is mainly ascribed to their redox properties, which have been shown to quench oxygen-derived free radicals by donating hydrogen atoms or electrons [5]. Biomolecules fulfilling many functions, such as flavonoids, have been shown to be highly effective scavengers of a broad spectrum of oxidizing molecules and inhibitors of lipid peroxidation [6].

The genus *Clerodendrum* of the family *Lamiaceae* (*Verbenaceae*) is diverse, comprised of 580 species of small trees, shrubs, lianas, or, occasionally, perennial herbs, most growing in tropical and subtropical regions [7]. The species *C. cyrtophyllum* Turcz, can be found on plains at altitudes below 1,700 m, on hills, in forests, and near trenches in Southern China. The richest wild population is found on Hainan Island. In Chinese, the plant it is called “da qing,” and it has been used in traditional Chinese medicine for the treatment of various ailments. The roots and leaves are used as anti-inflammatories, analgesics, and carminatives. *C. cyrtophyllum* has been used to treat colds, high fever, epidemic encephalitis, encephalitis B, migraines, hypertension, enteritis, dyspepsia, inflammation of the throat, rheumatic arthritis, carbuncles, furuncles, snakebites, and the plant has been used to decrease dampness [8]. In several studies, phenolic acids, polyketides, diterpenes, triterpenes, glucosides, proteins, and sterols have been isolated from various plant parts, and phenols comprise the major constituents of the plant [9], [10]. *C. cyrtophyllum* may be an excellent source of antioxidants: its methanolic extract has strong DPPH radical-scavenging activity [11]. However, the economic feasibility of industrial processing of *C. cyrtophyllum* requires more investigation to optimize the extraction process to increase the yield of extracted active substances.

Ultrasonic-assisted extraction (UAE) has been used to extract functional components from different matrixes; it is more rapid than conventional extraction techniques. UAE creates shear forces that break cell walls mechanically, simultaneously facilitating the release of cellular constituents of plant material into the extraction solvent without chemical degradation [12]. However, the efficiency of the extraction process is less than desireable [13].

An appropriate experimental design is necessary for any optimization study, and the two most common designs are single-factor experiments and response-surface methodology (RSM). Single-factor experiments were used here to provide data regarding extraction factors with significant effects on phenolic antioxidants from *C. cyrtophyllum* leaves. Next, these factors were analyzed by RSM for central composite rotatable design (CCRD) to more precisely determine optimal extraction conditions.

Continuing our ongoing research into natural plant antioxidants, we report a method of optimal antioxidant extraction [14], [15]. Our extraction parameters were ethanol concentration, extraction time, and extraction temperature. Phenolic compounds contain many hydroxyl groups and phenyls, so ethanol, a binary solvent, and water were critical extraction components. Our goal was to extract useful components from the leaves of *C. cyrtophyllum* while retaining optimal total phenolic content (TPC), total flavonoid content (TFC), and scavenging activity on 2,2′-diphenyl-1-picrylhydrazyl (DPPH) and 2,2′-azino-bis (3-ethylbenzothiazoline- 6-sulphonicacid) (ABTS).

## Materials and Methods

### Sample collection and pretreatment

Leaves of *C. cyrtophyllum* were collected at a very limited scale (2 kg) surrounding the Dead Crater Garden on Hainan Island, China. The People's Republic of China issued the specific permissions are required from authority of plant collection in a protected area of land, but not a national geological garden. The location we collect our plant materials is a national geological garden and the author was not obliged to have any permissions. This work did not involve endangered or protected species, the species *C. cyrtophyllum* is a common plant growing nearby the curbside. Leaves were selected, washed thoroughly in potable water, and then dried for 36 h using a hot air oven at 60°C. Dried leaves were then powdered using a herb disintegrator (118 Swing, Zhejiang, China) and subsequently sieved (20 mesh).

### UAE with *C. cyrtophyllum*

Ultrasonic-assisted extraction (UAE) were performed in in a digitally controlled ultrasonic device (Model XO-5200DTD, 200 W, 40 kHz; Nanjing Xian'ou Instruments Manufacture Company Ltd., China). Working frequency was fixed at 40 kHz. The extraction variables were selected according to Thoo *et al* [16]. Dried leaves of *C. cyrtophyllum* (5 g) were extracted twice with the required solvent, temperature, and time. Extracts were then filtered and the filtrate was prepared with a constant volume (250 ml) using 60% ethanol for estimation of phenolics and antioxidant measurements through various chemical assays. Each extraction was performed in duplicate and all analyses were performed in triplicate.

### TPC measurement

TPC from leaf extracts was measured according to the Folin-Ciocalteu (FC) procedure [17] as described with some modifications. The FC phenol reagent was prepared according to King's method [18]. Thus, 10 g sodium tungstate and 2.5 g sodium molybdate were gently dissolved in 70 mL deionized water, 5 ml 85% phosphoric acid, and 10 mL concentrated hydrochloric acid were subsequently added and allowed to reflux for 10 hr. Then, 1.5 g lithium sulfate and 6 mL hydrogen peroxide were added and refluxed for another 15 min until the color changed to a glassy yellow. The volume of the reaction mixture obtained was increased to 100 ml (q.s., deionized water) before usage. Then, 2 mL of diluted extracts were mixed with 2 mL of FC reagent. After 3 min, 750 μL of sodium carbonate anhydrous solution (7.5%, w/v) was added and the sample was vortexed. The absorbance at 765 nm versus a blank control was measured with a UV light spectrophotometer (Shimadzu UV2754) after a 2-h incubation in the dark at room temperature. Measurements were calibrated to a standard curve of prepared gallic acid solution ranging from 0–100 µg/mL with *y* = 0.0480x – 0.0071 (*R* ^2^ = 0.9991) and TPC was then expressed as mg of gallic acid equivalents (GAE) per g of dry weight (DW).

### TFC measurement

Estimation of TFC in extracts was performed according to colorimetric method [19] with some modifications. The reaction mixture contained 1.0 mL of extract, 4 mL of 60% ethanol and 0.3 mL of 5% sodium nitrite. Six minutes later, 0.3 mL of 10% aluminium nitrite was added. In the next six minutes, 4 mL of 1 M sodium hydroxide solution were added and the volume was increased to 10 mL (q.s. 60% ethanol). Immediately, the reaction mixture absorbance was measured by a spectrophotometer at 510 nm against a blank (control) and used to calculate TFC using rutin as a standard *y* = 0.0118x+0.0023, (*R* ^2^ = 0.9995). The linear relationship between absorbance and flavonoids content ranged from 15–75 µg/mL. TFC was then expressed as rutin equivalents (RE), in mg RE per g DW.

### DPPH radical scavenging capacity measurement

The radical scavenging ability of 2,2'-diphenyl-b-picrylhydrazyl (DPPH) was estimated by a method adapted from Sharififar *et al* [20]. Thus, an aliquot of extract (0.1 mL) was added to 3.9 mL of ethanolic DPPH (60 µM). The mixture was shaken vigorously and left to stand at room temperature for 30 min in the dark and absorbance was measured at 517 nm. The free radical scavenging activity was calculated as follows:
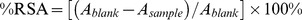
where *A_blank_* was the absorbance of the control reaction (containing all reagents except the test compound), and *A_sample_* was the absorbance of the test compound.

### ABTS radical scavenging capacity measurement

Free radical scavenging capacity using a stable ABTS radical was performed according to Thoo *et al* [16]. with some modifications. The ABTS radical solution was produced by gently mixing 10 mL of 7 mM ABTS solution and 10 mL of 2.45 mM potassium persulfate solution. This was allowed to stand in the dark at room temperature for 12–16 h. The ABTS radical solution was adjusted with ethanol to an absorbance of 0.7 (±0.02) at 734 nm before usage. Extract (100 µl) or ethanol (100 µl, control) was added to 3.9 mL ABTS radical solution and allowed to react for 30 min until a stable absorbance was obtained. The decrease in absorbance at 734 nm was measured against a blank (ethanol). Antioxidant activity of ABTS radical scavenging capacity was calculated as a scavenging percentage:
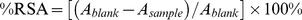
where *A_blank_* was the absorbance of the control reaction(containing all reagents except the test compound), and *A_sample_* was the absorbance of the test compound.

### RSM experimental design

A five-level, three-variable central composite rotatable design [21] was developed to determine the best combinations of extraction conditions for TPC from *C. cyrtophyllum* leaves. Three independent variables selected were ethanol concentration, extraction temperature, and extraction time. From single-factor experiments, the range for each independent variable was preliminarily determined and later used in subsequent experiments to test additional independent variables. Eight factorial points were used, six axial points (two axial points on the axis of each design variable at a distance of 1.68 from the design center), and four center points leading to 18 experimental runs. The actual and coded levels of the independent variables used in the experimental design are shown in Table 1. Yield values of TPC, TFC, DPPH and ABTS radical-scavenging capabilities of *C. cyrtophyllum* leaf extract were evaluated with multiple linear regression to fit the following empiric second-order polynomial model.
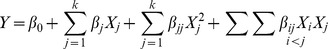
where *Y* represents the response function; *β_0_* is an intercept and*β_i_*, *β_ii_*, and*β_ij_* are the regression coefficients of the linear, quadratic, and interactive terms, respectively; accordingly *X_i_*, *X_i_^2^*, and *X_i_X_j_* represent the coded independent variables, respectively; *k* is the number of variables.

**Table 1:** Five-level, three-independent variable central composite rotatable design and experimental data for response variables for optimization of *C. cyrtophyllum* leaf extracts.

Run	Process variables – real and (coded) values			Responses^a^			
	*X_1_*, EtOH (%)	*X_2_*, Time (min)	*X_3_*, T (°C)	TPC (mg GAE/g DW)	TFC (mg RE/	DPPH radical- scavenging capacity (%)	ABTS radical- scavenging capacity (%)
1	20 (−1)	60 (−1)	50 (−1)	6.6±0.1	14.3±0.2	54.0±1.0	55.9±1.8
2	20 (−1)	60 (−1)	70 (1)	11.0±0.1	20.3±0.4	72.9±1.2	87.6±2.7
3	20 (−1)	100 (1)	50 (−1)	7.8±0.1	21.8±0.8	61.0±0.9	73.2±3.0
4	20 (−1)	100 (1)	70 (1)	12.2±0.3	25.2±0.7	76.4±1.3	88.0±1.5
5	60 (1)	60 (−1)	50 (−1)	13.6±0.3	41.5±0.4	76.5±0.7	89.2±0.7
6	60 (1)	60 (−1)	70 (1)	15.0±0.6	44.0±0.3	78.7±0.2	90.0±2.0
7	60 (1)	100 (1)	50 (−1)	14.8±0.5	42.5±0.8	79.9±0.4	91.1±1.3
8	60 (1)	100 (1)	70 (1)	16.2±0.2	48.1±1.5	81.7±0.5	87.9±0.5
9	74 (1.68)	80 (0)	60 (0)	15.9±0.5	46.1±0.2	77.2±0.5	89.6±0.7
10	6 (−1.68)	80 (0)	60 (0)	7.3±0.3	12.7±0.6	55.9±1.2	66.1±0.6
11	40 (0)	114 (1.68)	60 (0)	12.7±0.6	36.2±0.6	76.6±0.6	89.0±0.2
12	40 (0)	46 (−1.68)	60 (0)	11.2±0.2	30.0±0.5	71.2±0.9	80.7±1.1
13	40 (0)	80 (0)	77 (1.68)	14.6±0.5	35.0±0.6	79.5±0.5	90.9±1.6
14	40 (0)	80 (0)	43 (−1.68)	11.0±0.1	27.8±0.4	63.1±1.7	80.6±1.3
15	40 (0)	80 (0)	60 (0)	14.8±0.8	42.8±0.3	82.8±0.3	89.4±0.8
16	40 (0)	80 (0)	60 (0)	15.3±0.3	40.7±0.6	83.8±1.1	91.0±0.7
17	40 (0)	80 (0)	60 (0)	15.0±0.6	41.4±0.5	83.5±1.1	90.1±1.0
18	40 (0)	80 (0)	60 (0)	15.0±0.8	40.8±0.3	85.6±0.7	91.8±0.5

a Responses are the means ± SD (*n* = 3).

### Statistical analysis

Results were expressed as mean ± standard deviation of replicate solvent extractions and triplicate of assays and analyzed by Statistical Analysis System (SAS, version 9.1). Data were analyzed by ANOVA (*p*<0.05). Optimal extraction conditions were estimated through three-dimensional response surface analyses of the three independent variables and each response variable.

## Results and Discussion

### Single-factor experiments

First, we investigated whether ethanol concentration, extraction temperature, and time could be optimized for phenolic and flavonoid antioxidant extraction using single-factor experiments to determine appropriate experimental ranges for subsequent analyses.

### Effects of ethanol concentration

**Figure 1:**
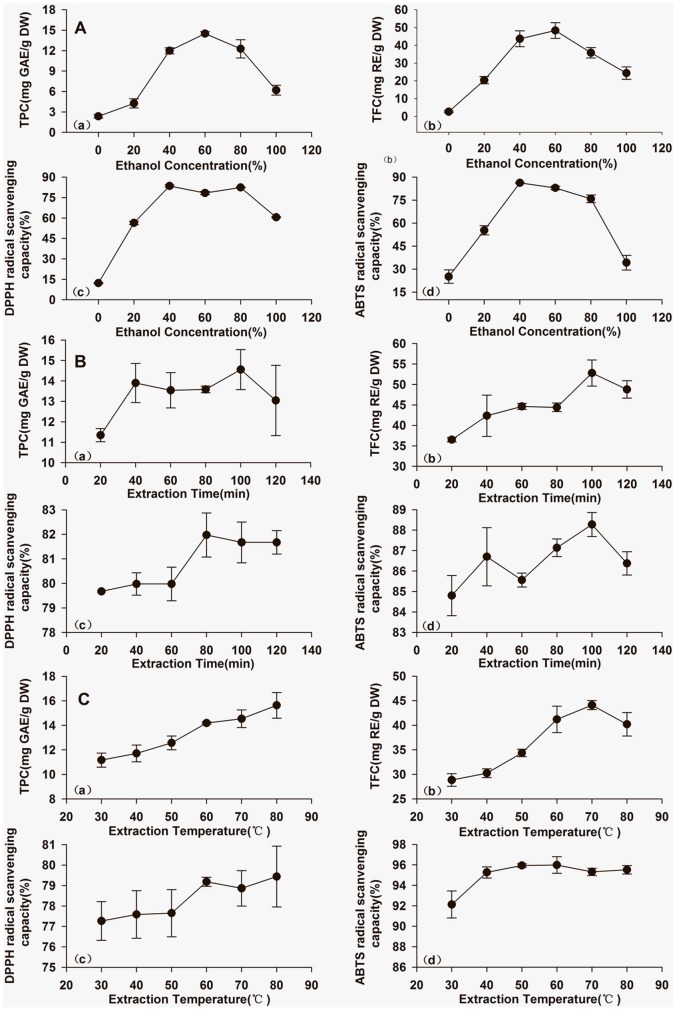
Effect of (A) ethanol concentration (extraction time 60 min, extraction temperature 60°C), (B) extraction time (40% ethanol, extraction temperature 60°C), (C) extraction temperature (40% ethanol, extraction time 80 min) on TPC, TFC, DPPH and ABTS radical scavenging capacity from *C.* *cyrtophyllum* leaf extracts.

Ethanol concentrations of 0%, 20%, 40%, 60%, 80% and 100% have been used extraction. Fig. 1A depicts of phenolic yield extracted (TPC and TFC) and the antioxidant capacities of those agents (against ABTS and DPPH radicals), and shows that both were greatly influenced by ethanol concentration. TPC and TFC recovery was parabolic with a maximum value at 60% ethanol. This was followed by a considerable decline with greater concentrations of ethanol. ABTS radical-scavenging capacities initially increased and peaked at 40% ethanol, then decreased considerably. DPPH radical-scavenging capacity declined with further increases in ethanol after the maximum values, 40% and 80% ethanol, respectively, were reached.

We found that TPC, TFC, and antioxidant capacities were not optimized when extracted with pure water or 100% ethanol, a finding that agrees with previous reports which suggest that a binary solvent system was superior to a mono-solvent system when extracting phenolic antioxidants and preserve their relative polarity [16], [22]. Similar polarity was detected for the phenolic and flavonoid components of *C. cyrtophyllum* leaves. Maximum recovery was observed at 60% ethanol. These components are highly soluble in hydroalcoholic solutions, especially when in a glycoside form, which may explain recovery variations. More flavonoids were recovered than phenols. This could be because flavonoids comprise a majority of the total phenols. The remainder of the plant's metabolic flavonoids are glycosides and derivatives with non-phenolic hydroxyl groups.

Antioxidant capacity was sensitive to solvent polarity, and a single ethanol concentration recovered all individual phenolic and antioxidant plant compounds. Extracts obtained at low ethanol concentrations (40%) had a greater scavenging capacity for both DPPH and ABTS radicals. Antioxidant contents of *C. cyrtophyllum* are more hydrophilic, so high-ethanol solvents may solubilize more lipophilic compounds. Briefly, a high yield of individual phenolic compounds does not necessarily indicate maximal antioxidant capacity. Phenolic compounds with the best antioxidant capacities were of intermediate polarity and their solubility was sensitive to solvent polarity.

Because excessive quantities of solvent can compromise the extraction of phenolic compounds and impair their antioxidant capacity, 40% ethanol was used for subsequent RSM to optimize extraction conditions [23].

### Effects of extraction time

Extraction time was important in obtaining phenolic extracts capable of scavenging DPPH and ABTS radicals. With 40% ethanol, extraction times from 20 to 120 min and an extraction temperature of 60°C were studied. As shown in Fig. 1B, the extraction time affected TPC and TFC significantly, but the antioxidant capability did not vary visibly. TPC and TFC yield from the extract and DPPH radical-scavenging capacity was enhanced with a longer extraction time, peaking at 100 min, after which values decreased slightly. This effect may be attributable to the time required for dissolution and diffusion of these compounds from the plant cell membrane into the solvent media by ultrasonic cavitation [12]. Recovery is also governed by the equilibrium concentrations achieved before their corresponding apparent reductions and rapid degradations [24].

The optimum extraction time for phenolic compounds and flavonoids, perhaps due to similar degrees of polymerization and solubilities of common phenolic flavonoids, required as much time as was needed to reach equilibrium between the solution in the plant material of *C. cyrtophyllum* as the bulk ethanol solution. Prolonging extraction times may allow recovered phenolic compounds to decompose.

The optimum extraction time for antioxidant compounds varied with antioxidant capacity. Antioxidant capacity, measured with ABTS, peaked at 80 min. Antioxidant capacities may not be solely attributable to scavenging a single group of radicals, but may be due to the scavenging of ABTS radicals, DPPH radicals, or both.

Because little differences were observed in phenolic yields extracted over 80 min and 100 min, even accounting for extraction efficiency and energy costs, an extraction time of 80 min was used for RSM.

### Effects of temperature

Heat can release large amounts of phenolic compounds in some cases, as described by Silva *et al* [23]. Here, incubation temperatures for phenolic antioxidant recovery were between 30–80°C (40% ethanol, 80 min extraction time). A direct relationship was observed between the extraction temperature and TPC recovery, as shown in Fig. 1C. With respect to TFC recovery, and ABTS and DPPH radical-scavenging capacity, the extraction temperature was optimal at 70, 60, and 60°C, respectively.

Increased temperature led to increases of cavitation bubble number, surface contact area and decreases of solvent media viscosity and density. These factors favored the release of phenolics from plant material and plant cell decomposition, enhancing solubility and diffusion coefficients [25]. According to the equilibrium principle, elevated temperature improved the extraction rate and reduced the extraction time required for maximum phenolic recovery. Increasing temperature may accelerate the transfer of phenolic compounds in *C. cyrtophyllum* and disrupt plant cellular constituents which may lead to increased cell membrane permeability. Also, elevated temperatures may not be suitable for all phenolic compounds, and higher proportions of thermally stable phenolic compounds might be more appropriate to extract under elevated temperatures. The TFC recovery was maximized at 70°C, an advantage likely offset by the decomposition of some thermally unstable flavonoids. Similar phenomena were observed with respect to antioxidant capacity, which peaked at 60°C, and then declined moderately with further increases in temperature. This may be ascribed to the denaturation of some thermo-sensitive non-phenolic antioxidants that can be mobilized at lower temperatures [26].

Considering the industrial efficiency requirements as well as accounting for inherent compromises between antioxidant yield and antioxidant capability, an optimal temperature of 60°C, was used for RSM optimization.

### Optimization by RSM

Central composite rotatable design (CCRD) was used to further optimize the extraction conditions with respect to the concentration of antioxidant compounds in *C. cyrtophyllum* leaf extracts. An ethanol concentration of 40% (v/v), an extraction time of 80 min, and extraction temperature of 60°C were chosen from previous single-factor experiments. The response values of TPC, TFC, DPPH, and ABTS radical-scavenging of extracts obtained under various experimental conditions are shown in Table 1. Maximum recovery of TPC (16.2±0.2 mg GAE/g DW) and TFC (48.1±1.5 mg RE/g DW) was recorded during Run No. 8, and maximum radical-scavenging capacity of DPPH (85.6±0.7%) and ABTS (91.8±0.5%) were recorded during Run No. 18. The lowest TPC (6.6±0.1 mg GAE/g DW), DPPH (54.0±1.0%) and ABTS (55.9±1.8%) radical-scavenging capacities were observed in Run No. 1. The lowest TFC (12.7±0.6 mg RE/g DW) was detected at Run No. 10.

### Fitting the model

Multiple regression analysis was performed based on the experimental data, and second-order polynomial models representing the recoveries of TPC, TFC, DPPH, and ABTS. The extract radical-scavenging capabilities as response variables are depicted in Table 2. Where possible, models were simplified by elimination of statistically insignificant terms (*P*>0.05).

**Table 2:** Regression models fitted to the experimental data of response variables.

Response	Model equationa	Probability of lack of fit	*R* ^2^
TPC (mg GAE/g DW)	Y = 15.013364+4.513759*X*_1_+0.923033*X*_2_+2.182736*X*_3_−3.334297*X*_1_^2^−3.004297*X*_2_^2^−2.129297*X*_3_^2^−2.185563*X*_1_ *X* ^3^	0.1597	0.9937^b^
TFC (mg RE/g DW)	Y = 41.384322+18.636959*X*_1_+3.438491*X*_2_+3.676343*X*_3_−11.249728*X*_1_^2^−7.534728*X*_2_^2^−9.244728*X*_3_^2^	0.1040	0.9877^b^
DPPH radical scavenging capability (%)	Y = 83.864603+10.942177*X*_1_+3.247395 *X* 2+8.151488*X*_3_−15.882841*X*_1_^2^−8.517841*X*_2_^2^−11.097841*X*_3_^2^−10.931425*X*_1_ *X*_3_	0.1594	0.9822^b^
ABTS radical scavenging capability (%)	Y = 90.535606+11.522496*X*_1_+3.896676*X*_2_+7.59029*X*_3_−12.494807*X*_1_^2^−5.484807*X*_2_^2^−4.584807*X*_3_^2^−7.517612*X*_2_ *X*_3_−17.610938*X*_1_ *X*_3_−6.433863*X*_1_ *X*_2_	0.0617	0.9729^b^

a X1, EtOH (%); X2, Time (min); X3, T (°C). Coded values. b P<0.001.

The quality of the fit of the model was expressed by the *R* ^2^ correlation coefficient, and its statistical significance was confirmed with an *F*-test. ANOVA of response values revealed that experimental data were correlated as depicted in Table 2. Calculated models were used to explain 99.37%, 98.77%, 98.22%, and 97.29% of the results with respect to TPC, TFC, DPPH, and ABTS radical-scavenging capabilities, respectively. Generally, fitting of exploration and optimization response surfaces may cause misleading results, unless the model exhibits a good fit [27]. Results were found to be significant (*P*<0.001), attesting to the goodness of fit of the models. *F*-values, which indicate lack of fit, were all insignificant (*P*>0.05), further confirming model validity. The results indicate that the models could predict recovery rates of TPC, TFC, DPPH, and ABTS radical-scavenging capabilities of *C. cyrtophyllum* leafs extracts quickly and efficiently when independent variables were within the ranges depicted here.

### Analyses of the regression coefficients and response surfaces

**Figure 2:**
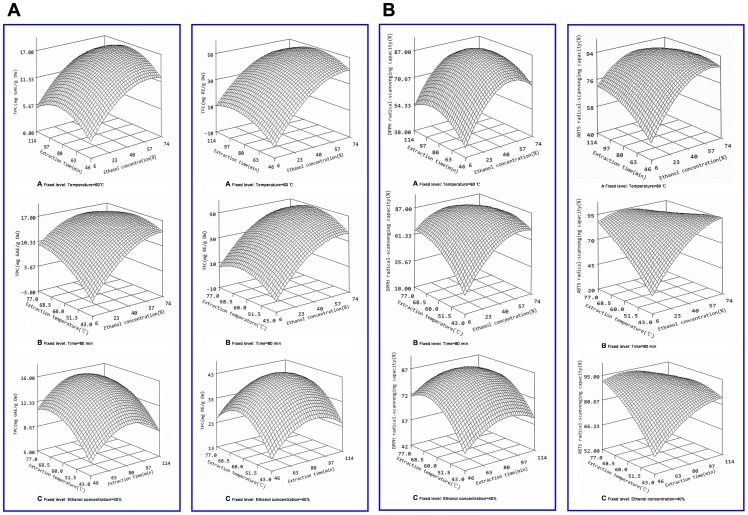
Response surface plots of TPCs of *C.* *cyrtophyllum* leaf extracts as affected by ethanol concentration, temperature, and time in UAE. (A) Ethanol concentration and time (temperature 60°C); (B) ethanol concentration and temperature (time 80 min); (C) temperature and time (40% ethanol).

Regression coefficients of the models for TPC, TFC, DPPH, and ABTS radical-scavenging capacities obtained by the multiple linear regressions are shown in Table 2. Variables in their coded form permitted a direct interpretability of variation in the linear, quadratic, and interaction effects of the independent variables. Three-dimensional response surface plots (Fig. 2) were constructed to predict the effects of the independent variables and their mutual interaction on the response variables. The surface plots facilitated the visualization of statistically significant factors derived from the statistical analysis. The plots were generated by plotting the response using the z-axis against two independent variables while keeping the remaining independent variable at zero level.

Regarding the ethanol concentration (*X*_1_), linear effects were confirmed to be statistically significant for TPC, TFC, DPPH, and ABTS radical-scavenging capacities, as indicated by the *P* value. A negative quadratic effect of *X*_1_ was observed with respect to the response variables, indicating that the response variables peak at a certain ethanol concentration, then started to decrease with further increases in ethanol. As shown in Fig. 2 (A, B), ethanol concentration influenced responses more significantly than temperature and time (optimal ethanol concentration ∼50–60%). This might be due to the solvent polarity which is suitable for phenol-enriched extraction. Similar results [28] have also been reported for the extraction of phenolic antioxidants from wheat. Maximum recovery was observed at 50% and 60% EtOH.

For extraction time (*X*_2_), all response variables exhibited significant, linear, negative quadratic effects. The fact that the effects of *X_2_* were statistically significant and linear indicated that increased extraction time improved antioxidant recovery. The fact that the effects of *X_2_* were negative and quadratic confirmed the deceleration in the extraction recoveries after equilibrium was reached. In this way, excessive time was found to be not useful for extraction of more antioxidants. The effects of extraction time combined with those of the two other factors on the recovery of TPC, TFC, DPPH, and ABTS radical-scavenging antioxidants are shown in Fig. 2 (A, C). Under each condition, extraction recoveries increased with increasing extraction time from 46 to ∼80 min, but extraction times over 86 min appeared diminish extraction yield. This indicated that extraction times between 80–86 min had a marked effect on response.

For the temperature of extraction (*X*_3_), a linear effect was detected for all response variables, confirming that increased temperature improves the solubility and diffusion coefficients of antioxidants and allows greater recovery. The effects of *X*_3_ were negative and quadratic, indicating the degradation of thermo-sensitive antioxidants at temperatures beyond a certain upper limit. The effects of extraction temperature on each of the other two factors on the response variables showed similar patterns of extractability, as shown in Fig. 2 (B, C). The response values increased to a certain value as temperature increased from 43°C to 63°C, and decreased thereafter.

The cross-effect between ethanol concentration × temperature (Fig. 2A), ethanol concentration × time (*X*_1_×*X*_3_) (Fig. 2B) and temperature × time (Fig. 2C) were proved to be negative for all response variables, which may be attributable to the poor solubility of some of the antioxidants at high ethanol concentration and to degradation of antioxidants after long extractions and at high temperatures.

### Experimental validation of optimal conditions

To verify the predictive capacity of the model, experimental confirmation was performed using the optimized conditions obtained depicted in Table 3. Measured values were consistent with values predicated by the model equation. The strong correlation observed confirmed the predictability of the response models for the evaluation of the TPC, TFC, DPPH, and ABTS radical-scavenging capabilities of *C. cyrtophyllum* leaves and confirmed that the response model could adequately reflect the expected optimization.

**Table 3:** Predicted and experimental values of response variables under optimal conditions.

Responses	Optimum extraction conditions			Maximum value	
	EtOH (%)	Time (min)	T (°C)	Experimental^a^	Predicted
TPC(mg GAE/g DW)	60.9	85.4	63.3	16.8±0.2	16.7
TFC(mg RE/g DW)	67.7	82.9	63.0	49.3±0.4	49.4
DPPH radical scavenging ability (%)	48.8	85.1	63.9	86.8±0.3	86.4
ABTS radical scavenging ability (%)	50.6	81.3	63.4	92.9±0.5	93.2

a Responses are the means ± SD (n = 3).

### Correlation analyses

ANOVA was used to estimate the statistical significance of the correlations between the response variables of TPC, TFC, and their radical-scavenging activities with respect to different extraction conditions. Correlation coefficients (*R* ^2^) between TPC and TFC, TPC and DPPH, TPC and ABTS, TFC and DPPH, and TFC and ABTS are depicted in Table 4 (*P*<0.05). Thus, the extraction of antioxidants from *C. cyrtophyllum* leaves was influenced by ethanol concentration, and this it may have been associated with bioactive phenolic flavonoids, which comprise a majority of the total phenols. In accordance with several previous studies, significant (*P*<0.05) and positive correlations were observed between ABTS and DPPH radical-scavenging capacity (0.7617), indicating that these two methods had similar predictive ability with respect to the antioxidant capacities of extracts from *C. cyrtophyllum* leaves and ethanol concentration [16].

**Table 4:** Correlation between response variables under different extraction conditions.

*r* ^2^	EtOH (%)			Time (min)			*T* (°C)		
	TPC	TFC	DPPH	TPC	TFC	DPPH	TPC	TFC	DPPH
TFC	0.9185^a^			0.6031^NS^			0.8329^c^		
DPPH	0.7537^c^	0.8763^b^		0.2258^NS^	0.5413^NS^		0.9375^a^	0.8101^c^	
ABTS	0.8162^c^	0.8464^b^	0.7617^c^	0.7318^c^	0.6486^NS^	0.5449^NS^	0.3463^NS^	0.3599^NS^	0.3121^NS^

a *P*<0.005, ^b^ *P*<0.01, ^c^ *P*<0.05; NS: non-significant; *r*: correlation coefficient.

However, with respect to extraction time, phenolic compounds were only moderately positively correlated with antioxidant activity. Only one substantially significant correlation was observed between TPC and ABTS (0.7318) at *P*<0.05. This result was consistent with a previous report showing that some bioactive compounds with ABTS radical-scavenging capacity may not exert DPPH radical-scavenging capacity [29].

Strong correlations were observed between DPPH, TPC, and TFC at a variety of extraction temperatures, as shown in Table 4. A slight but positive correlation was observed between ABTS and TPC, TFC and DPPH. These experimental results show that the phenolic antioxidants extracted from *C. cyrtophyllum* leaves might have different levels of thermostability. Increased temperature may improve extraction of thermally stable phenolic compounds responsible for the elimination of DPPH radicals but may permit decomposition of ABTS radical-scavengers. Thus, the type of antioxidant capacity desired will inform the selection of the optimal extraction temperature.

## Conclusion

From our single-factor experiments with antioxidant extraction from *C. cyrtophyllum* leaves, RSM could optimize the extraction process. A second-order polynomial model satisfactorily described the experimental data. The optimum extraction conditions are depicted in Table 3. Extraction variables were significantly correlated with yield (*P*<0.05), especially regarding ethanol concentration, which was the most important factor in the extraction process. Phenol and flavenoid concentrations were significantly correlated with radical-scavenging capacity with respect to ethanol concentration. Thus, our work provides a high-yield technique for antioxidant extraction from *C. cyrtophyllum* for the food and alternative/complementary medicine industry. Future studies to identify the predominant antioxidant compounds present in *C. cyrtophyllum* and mechanisms of antioxidant activity are warranted.
